# Predicting the Severity of Lockdown-Induced Psychiatric Symptoms with Machine Learning

**DOI:** 10.3390/diagnostics12040957

**Published:** 2022-04-12

**Authors:** Giordano D’Urso, Alfonso Magliacano, Sayna Rotbei, Felice Iasevoli, Andrea de Bartolomeis, Alessio Botta

**Affiliations:** 1 Section of Psychiatry, Department of Neuroscience, Reproductive and Odontostomatological Sciences, University of Naples Federico II, 80131 Napoli, Italy; giordano.durso@unina.it (G.D.); felice.iasevoli@unina.it (F.I.); andrea.debartolomeis@unina.it (A.d.B.); 2 IRCCS Fondazione Don Carlo Gnocchi, 50143 Florence, Italy; 3 Department of Electrical Engineering and Information Technology, University of Naples Federico II, 80138 Napoli, Italy; sayna.rotbei@unina.it (S.R.); a.botta@unina.it (A.B.)

**Keywords:** machine learning, COVID-19, prediction, obsessive-compulsive disorder, depression, anxiety

## Abstract

During the COVID-19 pandemic, an increase in the incidence of psychiatric disorders in the general population and an increase in the severity of symptoms in psychiatric patients have been reported. Anxiety and depression symptoms are the most commonly observed during large-scale dramatic events such as pandemics and wars, especially when these implicate an extended lockdown. The early detection of higher risk clinical and non-clinical individuals would help prevent the new onset and/or deterioration of these symptoms. This in turn would lead to the implementation of public policies aimed at protecting vulnerable populations during these dramatic contingencies, therefore optimising the effectiveness of interventions and saving the resources of national healthcare systems. We used a supervised machine learning method to identify the predictors of the severity of psychiatric symptoms during the Italian lockdown due to the COVID-19 pandemic. Via a case study, we applied this methodology to a small sample of healthy individuals, obsessive-compulsive disorder patients, and adjustment disorder patients. Our preliminary results show that our models were able to predict depression, anxiety, and obsessive-compulsive symptoms during the lockdown with up to 92% accuracy based on demographic and clinical characteristics collected before the pandemic. The presented methodology may be used to predict the psychiatric prognosis of individuals under a large-scale lockdown and thus supporting the related clinical decisions.

## 1. Introduction

In the framework of a multi-modal conceptualisation of mental health, it is well known that environmental stressors might, in most cases, elicit the onset of psychiatric diseases in vulnerable individuals, or increase the severity of symptoms in psychiatric patients [1]. Starting in late 2019, the COVID-19 pandemic caused millions of deaths and severe physical complications in the global population. Indirect consequences of the health crisis as well as social restrictions and economic constraints led to psycho-social repercussions due to forced social distancing, disruption of stable behavioural patterns, and anxieties over the future. Indeed, several studies observed an increase in the incidence of psychiatric disorders in the general population [2,3] even if contrasting results have also been reported. For example, a higher risk for developing severe depression and anxiety symptoms has been found in respondents of an online survey with a self-reported history of mental health problems [4], as well as in respondents directly tested for the restriction of physical activity [5]. Patients with pre-existing psychiatric conditions have been further affected by reduced access to psychiatry and psychotherapy services, which probably contributed to the exacerbation of their symptomatology [6]. In fact, it has been reported that, during the pandemic, psychiatric patients exhibited higher anxiety, depression and stress levels with respect to the general population, and showed a significant increase in the frequency of suicidal thoughts and episodes of insomnia [7,8]. In this scenario, the early detection of the most vulnerable individuals among the general population and psychiatric patients would allow to prevent the onset or worsening of anxiety and depression symptoms, so reducing the burden of the pandemic on mental health and on national health systems. Several previous studies have used regression models to predict the evolution of psychiatric disorders. For example, Ready et al. used hierarchical regressions for predicting patients’ future substance use, social behaviours, risky behaviours, and psychological distress [9]. Furthermore, generalised linear regression was used for investigating the personality traits associated with depressive symptoms in the general population across a fifteen year period, and it has been proven that personality traits were poor predictors of depression for specific time points [10]. Similarly, Wang et al. [11] used an operating characteristic curve and logistic regression analyses and compared the accuracy of clinicians with the accuracy of regression on a standardised scale in terms of evaluating the risk of future suicide attempts in a sample of psychiatric residents. A recent investigation [12] applied multiple logistic regression analyses to identify the socio-demographic and clinical factors associated with depression in a large sample of patients in the context of a first episode psychosis program. The same method was used for identifying the predictors of depression and anxiety in Brazil during the initial outbreak of COVID-19 and it has been observed that females, younger adults, and individuals with fewer children had a higher likelihood of developing depression and anxiety symptoms [13]. During the past two decades, machine learning (ML) [14,15,16] has become one of the most well-known methods used for several purposes, including prediction. It can identify complex patterns and trends and find complicated correlations between variables. ML methods can easily handle multi-dimensional datasets and identify the most significant input variables for predicting outputs. Here, we propose an ML approach for identifying the demographic and clinical characteristics of individuals with a higher risk of a poor psychological outcome during the COVID-19 pandemic-related lockdown. We tested this approach on a sample of healthy individuals and on two different populations of psychiatric patients, i.e., patients with obsessive-compulsive disorder (OCD) [17] and patients with adjustment disorder (AD) [18], whose demographic and clinical information was collected before and during the Italian lockdown. Albeit the aim of the present work was not to draw conclusions about specific clinical issues, we selected these two psychiatric populations because we hypothesised that they could have been affected by the lockdown more than others. In fact on one hand, OCD patients often suffer from obsessive thoughts of contamination which could have been exacerbated by the risk of COVID-19 contagion. In addition, compulsive washing and cleaning, which are also typical OCD symptoms, could have been worsened by the continuous invitation of hand-washing and hygiene coming from governments and health authorities [19]. On the other hand, patients with AD are defined as having developed emotional or behavioural symptoms out of proportion to the severity or intensity of an identifiable stressor. We hypothesised that this could imply a greater vulnerability to the psychological impact of the lockdown. Since Italy was the first European country hit by the pandemic and the first to enact large-scale lockdown measures, people’s perception of the restrictive measures was probably more dramatic than anywhere else. Starting from this unique observatory, the aim of this study was to implement a tool which is able to provide prognostic elements that could support clinical decisions during large-scale lockdowns. In particular, the practical application of this tool would be the development of a risk scoring protocol used on a routine basis by clinicians and service providers for the primary and secondary prevention of psychiatric symptoms.

The contributions of this paper can be summarised as follows: (i) We created a methodology based on supervised machine learning to identify predictors of the severity of psychiatric symptoms during the Italian lockdown as well as to predict the degree of severity; (ii) We applied this methodology to a use case on a small sample of individuals showing how it can be used in a real case; and (iii) We presented preliminary results showing that our models are able to predict depression, anxiety, and obsessive-compulsive symptoms during the lockdown with up to 92% accuracy based on demographic and clinical characteristics collected before the pandemic.

The rest of the paper is organised as follows. The methodology, the data it is based on, how data are pre-processed, and how the methodology is evaluated are reported in Section 2. Section 3 illustrates the application of the methodology to a case study using a real dataset. A discussion of the results obtained in the case study is then reported in Section 4. Concluding remarks are drawn in Section 5.

## 2. Materials and Methods

### 2.1. Participants

We enrolled two convenience clinical samples (one involving OCD patients and one involving AD patients) and one sample of healthy subjects. All patients were being treated at the University Hospital Federico II of Naples (Italy). Eligibility criteria for patients were: 18–70 years of age; and psychopathological stability at baseline evaluation. Eligible healthy subjects were 18–70 years of age without history of psychiatric illness. They were selected in order to obtain mean values of their demographic variables (i.e., age, sex, education) comparable to those of clinical samples. Exclusion criteria were: having COVID-19 (even suspected); being exempt from quarantine for any reason; being hospitalised or having been hospitalised during the lockdown; having a severe chronic medical disorder. The demographic characteristics of participants are reported in the Supplementary material.

All procedures were approved by our institutional review board and in accordance with the Declaration of Helsinki and its later amendments. All participants provided their informed consent.

### 2.2. Clinical Measures and Data Collection

Demographic data (e.g., age, sex, education, profession) and information about medical history (e.g., psychiatric diagnosis, family history of psychiatric diseases, medical comorbidities) were collected. Moreover, the scores of two to four psychiatric scales were gathered from medical records or through telephone calls, depending on the group.

The Yale–Brown Obsessive-Compulsive Scale (Y-BOCS), a clinician-administered rating scale, assesses the current and lifetime presence of OCD symptoms and their severity the week before evaluation. It consists of a symptoms checklist with 54 obsessions and compulsions dichotomously scored as present or absent and of a rating scale assessing the severity of the current symptoms in terms of time spent, interference, distress, resistance, and control. The rating scales comprise 10 items: 5 for obsessions and 5 for compulsions. All items have a Likert-type scale ranging from 0 to 4 so that it is possible to obtain a total score of 0–40 for the overall obsessive-compulsive symptoms and two subtotal scores of 0–20 for obsessions and compulsions separately [20]

The Brown Assessment of Belief Scale (BABS) is a seven-item clinician-administered semi-structured scale designed to assess the degree of conviction and insight that patients have concerning their beliefs. It consists of 7 items: the first 6 items were added to obtain the total BABS score, while an additional item (ideas of reference) is not included in the total score. Each item is rated from 0 (non-delusional, or the least pathological) to 4 (delusional, or the most pathological) [21].

The Beck Depression Inventory-II (BDI-II) is a multiple-choice self-report inventory that consists of 21 items assessing the affective, cognitive and physical symptoms of depression. Each item is rated from 0 to 3 (from the least to the most severe) [22]. The State-Trait Anxiety Inventory-Y (STAI-Y) is a commonly used measure of trait and state anxiety. Form Y, its most popular version, has 20 items for assessing trait anxiety and 20 for state anxiety. All items are rated on a 4-point scale (e.g., from “Almost Never” to “Almost Always”). Higher scores indicate greater anxiety [23].

**Figure 1:**
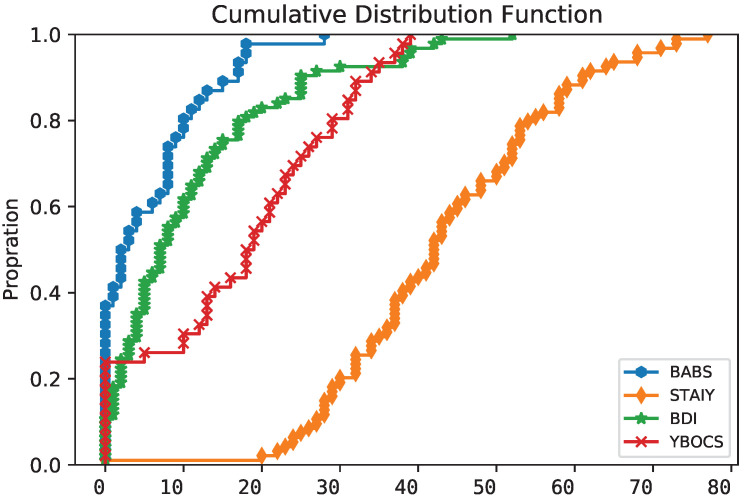
Cumulative distribution function of all scales at T0.

Figure 1 shows the cumulative distribution function of all scales. Note that BDI-II and STAI-Y are related to all three groups, whereas the Y-BOCS and the BABS are only related to the OCD patients because they are not meaningful for non-OCD individuals.

In line with the predictive purpose of this study, all scales were administered at two different time points, i.e., before the pandemic (baseline) and during the Italian lockdown period (follow-up), namely from March to June 2020. The baseline scores of the OCD and AD samples were gathered from the patients’ clinical records (STAI-Y and BDI-II for both groups, Y-BOCS and BABS for the OCD group) since the aforementioned clinical scales are collected on a routine basis. Given that patients could not be reached in person during the lockdown, the follow-up assessment was performed through a telephone call by an expert examiner blinded with respect to the patient’s diagnosis. For healthy subjects, STAI-Y and BDI-II scores at both time points were collected during the same telephone call with the baseline evaluation being retrospective in nature. The methodology worked on the values of these four scales administered before the lockdown and used such values as features (i.e., input values) for the prediction. The predicted value was then one of the rates of the same scales but during the lockdown. Basically, looking at the values of the scales before the lockdown together with other information regarding the participants (i.e., demographic data and information about medical history), properly trained ML algorithms can predict the values of any scale during a lockdown.

### 2.3. Data Pre-Processing

Before applying ML to the dataset described in the previous section, this dataset should be properly pre-processed. One of the critical requirements of an ML system is the ability to face and tackle the challenges of imperfect data (i.e., containing errors, noise, and missing values) [24]. In order to have uniform data, the dataset should be cleaned, pre-processed, and normalised. After gathering all the data, the scores of each questionnaire were calculated and added to the database. Thereafter, in order to make the prediction, the calculated scores were clustered into three to five groups. For the BABS scale, the scores were categorised into three groups: 0–5, 6–15, and >15. For the Y-BOCS, the categorisation by [25] was used: <7 likely to be sub-clinical, 8–15 mild OCD, 16–23 moderate OCD, 24–31 severe OCD, and 32–40 extreme OCD. The category used for BDI-II was: 0–13 for minimal depression, 14–19 for mild depression, 20–28 for moderate depression, and 29–63 for severe depression [22]. According to [26], for the STAI-Y scale, the possible categories were “no or low anxiety” (20–37), “moderate anxiety” (38–44), or “high anxiety” (45–80).

On one hand, it is important to underline that the individual and not the grouped scores were used as input for the prediction. On the other hand, the clustered values were used as the values to be predicted. For example, when classifiers predicted the BDI-II, the category of the scale of BDI-II as previously defined was used, and when the classifier predicted “mild depression”, it meant that the score was somewhere between 20 and 28. All missing values were replaced with “−1” and the whole feature set normalised.

### 2.4. Feature Importance

Before starting the classification, it is critical to know the importance of the features of the database. The dataset dimension can be reduced without losing information by knowing the importance of the features [27]. Another important outcome of the analysis of feature importance is the relation between the target variable and the features. This is an important aspect of the proposed methodology as it shows what are the important input parameters that count more for the prediction, the so-called predictors. The feature importance reveals what are the relations between the input and output parameters. In this research, the importance of the features was calculated and visualised for all analyses.

### 2.5. Prediction

**Figure 2:**
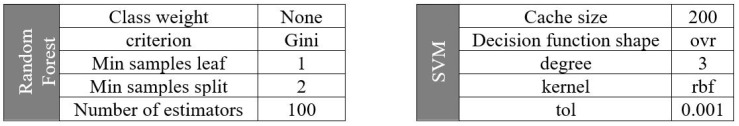
The parameters of models used in our methodology.

For the prediction of the individual condition in terms of psychiatric scales, two machine learning classifiers were used, the first being Random Forest. The idea behind Random Forest was to build multiple different decision trees. Then, it provided the best classification performance results from the results classified by each tree [28]. Random Forest is one of the group-based learning methods for solving classification and regression problems. In this method, multiple classifiers are used in group classification to obtain accurate results in comparison with a single classifier. Furthermore, in this method, the integration of multiple classifiers reduces the variance, particularly in the case of unstable classifiers, and can provide reliable results. Overall, Random Forest can manage many input variables without variable deletion, also it can compute the vicinity of pairs which is useful for locating outliers. It is resistant against outliers and noise, and able to diminish variance without increasing the bias of predictions [29]. The second classifier was the support vector machine (SVM). The SVM is an ML technique for building more complex models which allows one to convert input data into a high-dimensional space in order to find the best decision boundary between classes. [28,30]. It is one of the most popular and most widely used kernel-based learning algorithms. The SVM can define the best optimal hyper line for separating the dataset into a discrete number of predefined classes by using the training data. The SVMs use a section of the training set that is nearest to the best decision boundary in terms of feature space [29]. In Figure 2, all parameters of both models are depicted.

If the classifier uses the whole dataset for training the model, it is then impossible to evaluate the performance of such a model because the classifier can easily remember the correct answer, so the performance results are not reliable. The performance of the model should rather be evaluated on new data that have not previously been seen by the model. Thus, the dataset should be divided into two parts: the first part is used for model training and the second one for testing its performance [30].

In this research, in order to have more stable estimates and a more generalisable performance, the k-fold cross-validation method was used. The dataset was divided into k equal-sized subsets of individuals which were called folds. One of the k subsets was used for testing the performance of the model and the rest of the subsets (k-1 subsets) were used for model training. This procedure was repeated k times, changing the training/test set in each fold. In the end, each of the k subsets was used once as a test set for evaluating the model performance [31].

### 2.6. Performance Metrics

Four metrics were used for evaluating the performance of the classifiers in predicting the subject mental health during the COVID-19 lockdown. These metrics comprise precision, recall, F1-score, and accuracy. The number of false-positive (*FP*), false-negative (*FN*), true-positive (*TP*), and true-negative (*TN*) are used for obtaining the accuracy which is the ratio of correct predictions.

(1) $$Accuracy=\frac{TP+TN}{TP+TN+FP+FN}$$

The number of *FP*, *FN*, and *TP* are used for calculating the precision and recall which are defined as follows:

(2) $$Precision=\frac{TP}{TP+FP}$$

(3) $$Recall=\frac{TP}{TP+FN}$$

The F1-score is useful for unbalanced datasets [32]. It can be calculated by using the ratio of recall and precision or *TP*, *FP*, and *FN* as follows:

(4) $$F1=\frac{2*Precision*Recall}{Precision+Recall}=\frac{2*TP}{2*TP+FP+FN}$$

## 3. A Preliminary Study on a Real Dataset

In this section, we show how the methodology can be applied in a preliminary study. We highlight that the results are to be considered preliminary as the dataset is related to a small number of subjects.

### 3.1. Data Used

We used data from 94 subjects assessed at two different time-points, i.e., at baseline (before the pandemic) and at follow-up (during the COVID-19 lockdown). Of these subjects, 46 were OCD patients, 19 were AD patients, and 29 were healthy subjects. Baseline demographic and clinical characteristics of the three groups are reported in the Supplementary materials.

### 3.2. Results Obtained

The methodology was implemented and tested using the Python programming language. Four different case studies were performed for studying the impact of COVID-19 lockdown and any other kinds of lockdown on psycho-pathological patients and healthy individuals. In each of these analyses, the objective was to predict the status of target subjects during the first lockdown of COVID-19 measured through one of the scales. Moreover, for each scale to be predicted, we evaluated the importance of each feature for prediction in order to capture the variables (demographic, medical history, and psychiatric symptoms before the lockdown) that play a major role in the emergence of psychiatric symptoms during lockdown.

#### 3.2.1. Predicting Depression Symptoms

**Figure 3:**
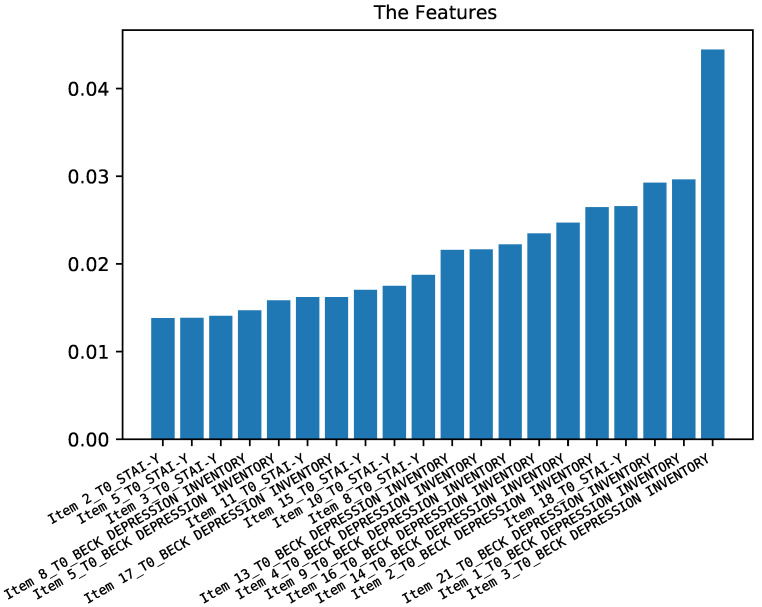
Importance of the different features for predicting depression symptoms.

In the first case study, the depression symptoms (measured through the BDI-II scale) during the first lockdown (T1) were predicted based on data gathered before such lockdown (T0). Figure 3 shows the correlation between BDI-II at time T1 and all other features at baseline (T0). This figure shows that the answers to the BDI-II and STAI-Y scales at time T0 played an important role with respect to other features.

**Figure 4:**
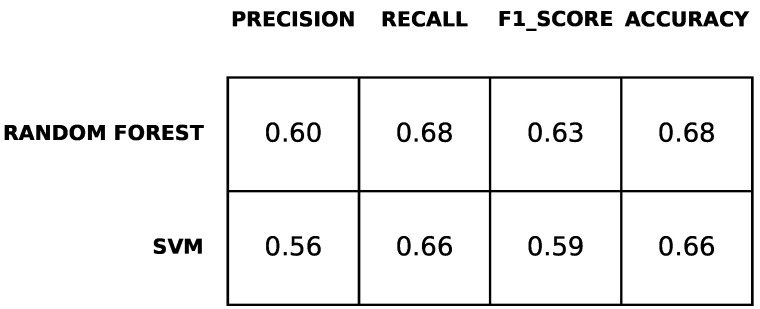
Results of the prediction of depression symptoms.

Figure 4 reports the performance measures of the prediction computed as described in Section 2. The results are obtained through a 10-fold cross-validation. The table shows that the classifiers predicted with accuracy up approximately 70% percent and F1-score up to approximately 63%. Precision and recall range between 55% and 68%. Furthermore, the table shows that the performance of both classifiers for predicting BDI-II at time T1 was approximately the same. The random forest classifier shows slightly better performance.

#### 3.2.2. Predicting Anxiety Symptoms

**Figure 5:**
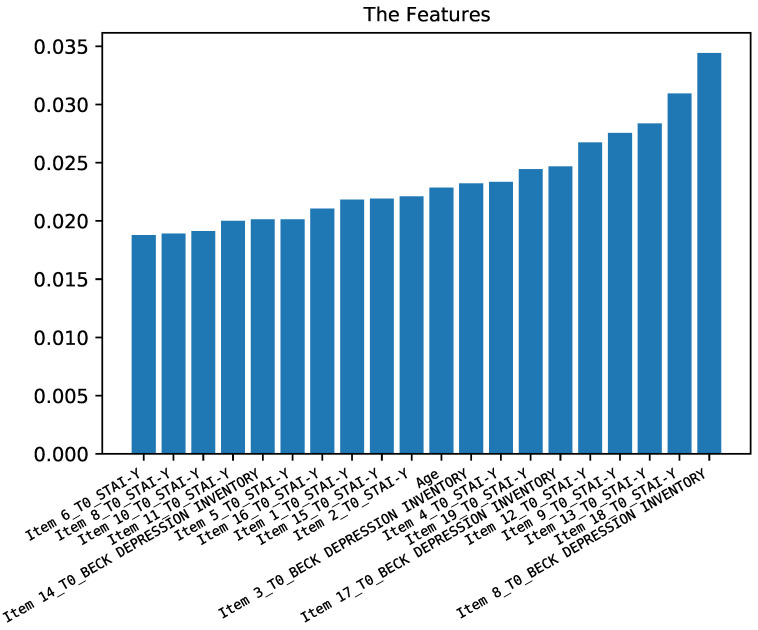
Importance of the different features for predicting anxiety symptoms.

The second case study was aimed at predicting the anxiety symptoms (measured through scale STAI-Y) at time T1 by using the rest of the features of the dataset including demographic data and the answers to the questionnaires of BABS, YBOCS, STAI-Y, and BDI-II at time T0. Figure 5 shows that the first 10 most important features are a combination of different items of BDI-II and STAI-Y at time T0, and age also plays an important role.

**Figure 6:**
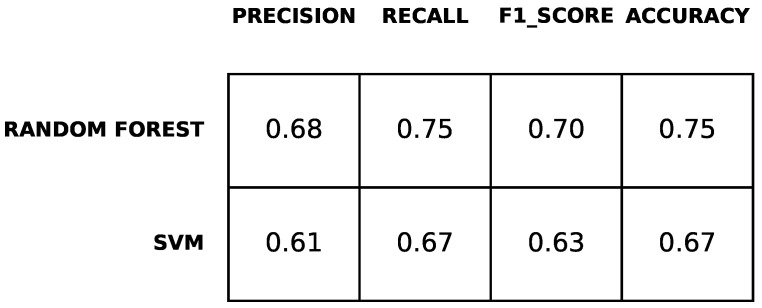
Results of the prediction of anxiety symptoms.

Figure 6 shows the performance of classifiers for predicting STAI-Y at time T1. As for the previous case study, (predicting BDI-II at time T1), random forest obtained a prediction performance higher than the support vector machine. The accuracy is up to 75% and F1-score is up to 70%.

#### 3.2.3. Predicting Obsessive and Compulsive Symptoms

**Figure 7:**
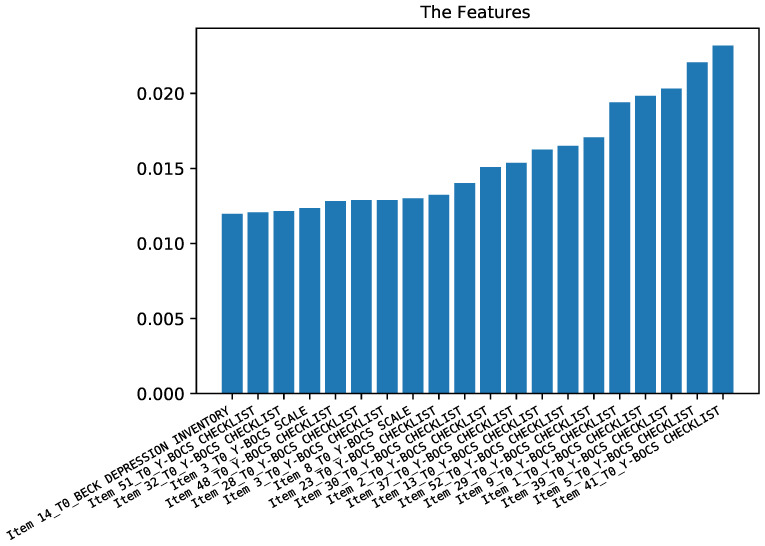
Importance of the different features for predicting obsessive and compulsive symptoms.

In the third case study, the obsessive and compulsive symptoms (measured through scale Y-BOCS) was the class to be predicted. Figure 7 shows the role of each feature in the prediction process. According to this figure, selected items of the scale Y-BOCS at time T0 are the most important ones.

**Figure 8:**
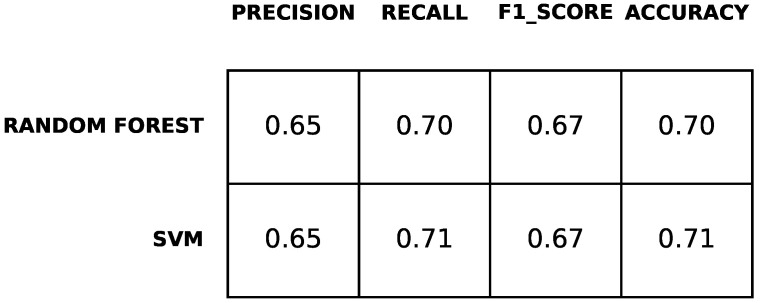
Results of the prediction of obsessive and compulsive symptoms.

According to Figure 8, the performance of ML classifiers for predicting the condition of subjects in terms of Y-BOCS values are approximately the same for both classifiers. The accuracy is up to 71% and the F1-score is up to 67%. The difference is mainly related to recall and accuracy. For random forest, it is approximately 70%, while for support vector machine, it is approximately 71%. Precision and F1-score are 65% and 67%, respectively.

#### 3.2.4. Predicting Belief Symptoms

**Figure 9:**
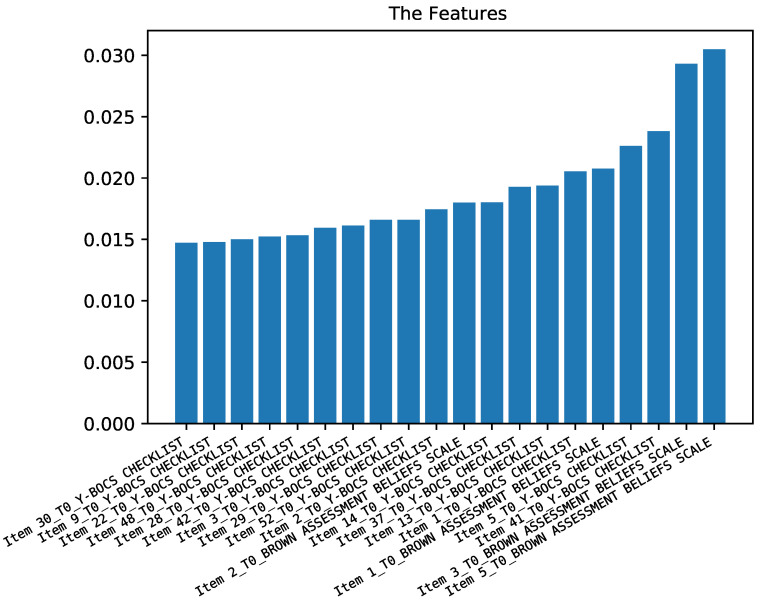
Importance of the different features for predicting belief symptoms.

In the last case study, belief symptoms at time T1 (evaluated through the BABS scale) is the class to be predicted. Figure 9 shows that there is a strong correlation between the scale BABS at time T1 with scales BABS and Y-BOCS at T0. These scales have a remarkable impact on the results in this case study.

**Figure 10:**
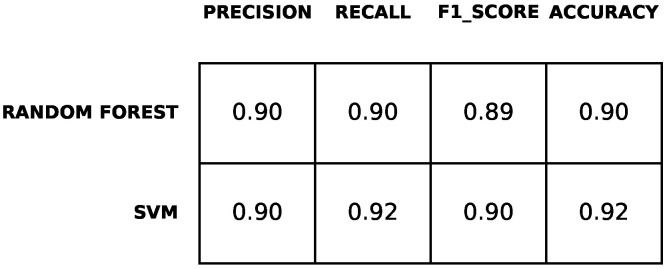
Results of the prediction of belief symptoms.

Figure 10 shows the performance of the two considered classifiers for predicting the scale BABS at time T1. The classifiers achieve accuracy values up to 92% and F1-score values of up to 90%. Comparing the two classifiers, precision values are mostly similar for both classifiers, while recall values are higher for the support vector machine.

## 4. Discussion

A few studies have used ML to predict the consequences of the COVID-19 lockdown in terms of psychiatric symptoms and to the best of our knowledge, none were based in Italy. Italy was the first European country to be hit by the COVID-19 pandemic and the first to enact an extended large-scale lockdown. Moreover, single regions such as Campania implemented additional restrictive provisions. This was an unexpected and unprecedented contingency, provoking a great amount of uncertainty about the future, psychological distress, worry, and anxiety in the population. In fact, a nation-wide lockdown and stay-at-home restrictions had never occurred in recent history in Europe before March 2020.

In the present study, we collected the demographic and clinical characteristics of healthy individuals and psychiatric patients from the Italian region of Campania before and during the COVID-19 lockdown with the aim of identifying possible predictors of the severity of psychiatric symptoms. We tackled this issue with two ML algorithms, i.e., Random Forest and the SVM. In a preliminary phase of this study, we also tested other algorithms including K-nearest neighbours and Naive Bayes, but Random Forest and SVM achieved the best performance. We set up a methodology based on these two algorithms that carefully processed the input data, clustering, cleaning, and normalising them, in order to identify which baseline variables were strongly associated with the clinical evolution at the follow-up.

Our models allowed us to identify the most predictive baseline features, therefore possibly increasing the knowledge about the interplay between specific clinical variables and/or between clinical and demographic variables. More importantly, our results showed that the severity of psychiatric symptoms such as depression, anxiety, and OCD during the lockdown could be predicted by taking into account items of the BDI-II, STAI-Y, and Y-BOCS collected at the baseline (as described in Section 3.2.1–Section 3.2.4) and no single predictor can be used for this task. In particular, the severity of anxiety symptoms, expressed as a total score on the STAI-Y collected during the lockdown, was best predicted by the score on some STAI-Y items and the score on a few BDI-II items gathered at the baseline. Similarly, the five most important features for predicting the BDI-II total score during the lockdown were the BDI-II items 3, 1, 21, 2, and item 18 of STAI-Y collected at the baseline.

Despite the prognostic potential of the described methodology and the opportunity to unravel unknown relations between clinically meaningful variables, the above results should be considered very cautiously because of the following limitations. First, we employed heterogeneous methods (i.e., medical records vs. phone calls) for collecting baseline data in psychiatric patients and healthy subjects. This difference makes it difficult to compare baseline findings in the three groups. Moreover, for healthy individuals, baseline and follow-up data were collected within a single telephone call during the lockdown. This might have affected pre-pandemic data reliability due to physiological oblivion as well as state-dependent memory recall and the selective attention biases of respondents. Furthermore, the results of the present study were based on a relatively small sample size and both clinical and non-clinical samples are probably not representative of the corresponding populations. Finally, since it is not possible to have baseline clinical information about healthy subjects (non-clinical by definition), we acknowledge that with respect to this set of data, the application of our methodology is just a hypothetical example of how to use baseline information to predict the psychiatric outcome of a given population during a lockdown. However, on the one hand, for healthy subjects, it is still possible to use demographic information—which is always available—to apply the described method and identify the most vulnerable subjects among the general population, which is of interest for public health policies. On the other hand, the baseline clinical information is already available for clinical populations and can be combined with demographic characteristics, so yielding potentially sensitive and useful predictors of psychiatric outcomes which can be used to set up prevention policies in psychiatric patients. In this view, our study might pave the way for future investigations aiming to practically apply the described models for the implementation of a risk scoring system. Such a system would allow clinicians and service providers involved in psychiatric prevention to enter into a computer the most appropriated indicators for the determination of the risk profile of a given individual and consequently enact personalised and well-timed therapeutical interventions. In our specific case of lockdown-induced psychiatric symptoms, these early interventions for at-risk subjects could consist in programs of more frequent tele-health psychiatric evaluations and/or psychotherapy sessions for those already in treatment and in the implementation of regular sessions of psychological support for healthy subjects.

The main purpose of our data analysis was to show how to practically apply the methodology on a real sample, and not to draw clinical conclusions. As a matter of fact, in the case of providing a larger sample for the algorithm, it would be possible to not only give more conclusive results but also explore the cases for which the algorithm provides accurate results and the cases for which the prediction is not accurate. Moreover, future studies could use this methodology to explore the predictive value of a greater number of variables with respect to those we considered, e.g., biochemical or imaging markers, which would increase the accuracy of the prediction.

## 5. Conclusions

In conclusion, here we propose an ML approach to identify the demographic and clinical variables suitable to predict the onset and/or worsening of psychiatric symptoms during a dramatic large-scale event such as the COVID-19 lockdown. In the present paper, we used the demographic and clinical information of two psychiatric populations (i.e., OCD and AD) and healthy individuals as case studies and demonstrated that the Random Forest and SVM models can predict the severity of depression, anxiety, and obsessive-compulsive disorder symptoms with an accuracy of up to 92% in each population. Should our preliminary findings be confirmed by future studies on larger samples, this model could be applied in cases of dramatic events causing large-scale lockdowns in two possible ways: in clinical settings, for the prevention of symptom deterioration in higher risk psychiatric patients; by health authorities, in order to implement public health programmes specifically targeting vulnerable clinical and non-clinical populations.

## Data Availability

Data available in an accessible repository on request.

## Supplementary Materials

The following supporting information can be downloaded at: https://www.mdpi.com/article/10.3390/diagnostics12040957/s1, Table S1: Demographic characteristics of the individuals used for the case study.
