# Supplementary material for: Predicting the Severity of Lockdown-Induced Psychiatric Symptoms with Machine Learning
Source: Diagnostics (Basel). 2022 Apr 12;12(4):957. doi: 10.3390/diagnostics12040957 (PMC9025309; doi:10.3390/diagnostics12040957)
Supplement: Supplementary file 1 [file diagnostics-12-00957-s001.zip › diagnostics-1649541-supplementary.pdf]

Predicting the Severity of Lockdown-induced Psychiatric Symptoms with Machine Learning

Giordano D'Urso [1], Alfonso Magliacano [2,*], Sayna Rotbei [3], Felice Iasevoli [1], Andrea De Bartolomeis [1], Alessio Botta [3]

[1] Section of Psychiatry, Department of Neuroscience, Reproductive and Odontostomatological Sciences, University of Naples Federico II, 80131 Napoli, Italy; giordano.durso@unina.it (G.D.); felice.iasevoli@unina.it (F.I.); andrea.debartolomeis@unina.it (A.d.B.)

[2] IRCCS Fondazione Don Carlo Gnocchi, 50143 Florence, Italy

[3] Department of Electrical Engineering and Information Technology, University of Naples Federico II, 80138 Napoli, Italy; sayna.rotbei@unina.it (S.R.); a.botta@unina.it (A.B.)

** Correspondence: a.magliacano@live.it*

Supplementary Table S1. Demographic characteristics of the individuals used for the case study.

SEX		Education				Family history of OCD		Medical comorbidities	
Female	Male	Primary	Middle	High	University	YES	NO	YES	NO
43	51	2	26	43	23	10	83	34	59

Age			Financial issues due to lockdown		Positive to CoViD-19		Health worker	
Age<26	25<Age<51	Age>50	YES	NO	YES	NO	YES	NO
15	46	33	6	85	15	78	4	89
